# Bovine Follicular Fluid Derived Extracellular Vesicles Modulate the Viability, Capacitation and Acrosome Reaction of Bull Spermatozoa

**DOI:** 10.3390/biology10111154

**Published:** 2021-11-09

**Authors:** Mohammad Mehedi Hasan, Qurat Ul Ain Reshi, Freddy Lättekivi, Janeli Viil, Kasun Godakumara, Keerthie Dissanayake, Aneta Andronowska, Ülle Jaakma, Alireza Fazeli

**Affiliations:** 1 Institute of Veterinary Medicine and Animal Sciences, Estonian University of Life Sciences, Kreutzwaldi 62, 51006 Tartu, Estonia; Mehedi.hasan@ut.ee (M.M.H.); qurat.reshi@ut.ee (Q.U.A.R.); kasun.godagedara@ut.ee (K.G.); keerthie.dissanayake@ut.ee (K.D.); ylle.jaakma@emu.ee (Ü.J.); 2 Department of Pathophysiology, Institute of Biomedicine and Translational Medicine, University of Tartu, Ravila St. 14b, 50411 Tartu, Estonia; freddy.lattekivi@ut.ee; 3 Department of Pharmacology, Institute of Biomedicine and Translational Medicine, University of Tartu, Ravila St. 19, 50411 Tartu, Estonia; janeli.viil@ut.ee; 4 Institute of Animal Reproduction and Food Research, Polish Academy of Sciences, Tuwima St. 10, 10-748 Olsztyn, Poland; a.andronowska@pan.olsztyn.pl; 5 Academic Unit of Reproductive and Developmental Medicine, Department of Oncology and Metabolism, The Medical School, University of Sheffield, Sheffield S10 2SF, UK

**Keywords:** extracellular vesicles, follicular fluid, spermatozoa, acrosome reaction, fertilization

## Abstract

**Simple Summary:**

Before the union of an egg and spermatozoon, several vital processes occur for fertilization in the female reproductive system. One of these processes is the maturation of spermatozoa which occurs in the female reproductive tract. Spermatozoa not undergoing maturation in the female reproductive tract are unable to penetrate the egg. Many reports have suggested the involvement of different factors in mediating the functional maturation of spermatozoa. Follicular fluid (FF) is named as one of those factors. FF is an ovarian fluid that plays an essential role in egg maturation and sources extracellular vesicles (EVs). EVs are nano-containers that are released from different cells and are present in all body fluids. Several studies have reported that FF supports the functional maturation of spermatozoa. Therefore, we hypothesized that FF EVs might have a role in inducing functional maturation in spermatozoa. Surprisingly, the FF-derived EVs were able to aid vital functional parameters of spermatozoa and the effects from EVs were species- and source-specific. Therefore, deciphering the cargo of FF EVs responsible for modulating spermatozoa’s functions can potentially prove beneficial in diagnosing and treating male infertility and improving the current assisted reproductive technology protocols.

**Abstract:**

While follicular fluid (FF) is known to enhance the functional properties of spermatozoa, the role of FF-derived extracellular vesicles (EVs) in this respect is unknown. We hypothesized that bovine FF EVs convey signals to spermatozoa supporting sperm viability, inducing sperm capacitation and acrosome reaction. In this study, the effects of bovine FF EVs on sperm functions are evaluated. Irrespective of the size of the follicles which FF EVs had originated from, they were capable of supporting sperm viability, inducing capacitation and acrosome reaction. These effects were specific to the source of bovine FF EVs, as human-cell-line-derived or porcine FF EVs did not affect spermatozoa viability or induced capacitation and acrosome reaction. A minimum of 5 × 10^5^ EVs/mL was adequate to maintain sperm viability and induce capacitation and acrosome reaction in spermatozoa. Interestingly, with FF EV trypsin treatment, FF EVs lost their ability to support sperm functions. In conclusion, this study demonstrates that bovine FF EVs can support spermatozoa function and may contribute to a favorable periconceptional microenvironment. This is an important aspect of the interactions between different sexes at the earliest stages of reproduction and helps to understand molecular mechanisms modulating processes such as sperm competition and female cryptic choice.

## 1. Introduction

The journey towards a successful pregnancy begins with the release of an oocyte along with follicular fluid (FF) [1]. FF is a complex and dynamic biological fluid derived from the plasma components that have crossed the blood–follicle barrier and metabolites secreted by granulosa and thecal cells [2]. FF serves as an important mediator of intercellular communication between somatic and germ cells of the ovarian follicles and also acts as a carrier of nutrients to the oocyte. FF is known to play a pivotal role in maintaining oocyte quality, inducing sperm capacitation, promoting fertilization and embryo development [3].

Following copulation, millions of spermatozoa start to ascend the maternal tract towards the oocyte; however, only from tens to hundreds of spermatozoa reach the oocyte and only one eventually fertilizes the oocyte. Spermatozoa survive the challenging environment of the female reproductive tract, bribe the immune system and undergo capacitation, hyperactivation and the acrosome reaction to be competent to fertilize the oocyte [4,5,6]. Upon arrival in the oviductal isthmus, highly selected spermatozoa maintain close contact with the endosalpingeal epithelium and stay quiescent until ovulation has taken place [7]. Upon induction of the capacitation, spermatozoa are released from the site of sperm storage and migrate towards the oocyte [8].

Capacitation involves a complex cascade of signaling mechanisms involving biochemical, biophysical and functional changes in the sperm plasma membrane and consequent increase in membrane fluidity, an efflux of cholesterol, redox regulation and tyrosine phosphorylation of proteins [9,10]. Capacitation induces hyperactivated motility and precipitates the acrosome reaction [11,12]. An acrosome reaction is an exocytotic event during which the sperm plasma membrane fuses with the outer acrosomal membrane leading to the release of acrosomal contents including hyaluronidase. A hyperactive acrosome-reacted spermatozoon can fully penetrate the zona pellucida, fuse with the oocyte, transfer the genetic material and thus fertilize the egg [13,14]. 

During ovulation, nearly 0.5% of the FF from the ruptured follicle enters the oviduct along with the ovum [15]. FF has been recognized as an important factor that enhances sperm capacitation and the acrosome reaction in various mammalian species including cattle [16], pigs [17], hamsters [18] and humans [1]. The majority of these investigations have implied that progesterone in the FF is responsible for the enhancement of sperm function and induction of acrosome reaction [19,20,21]. Several recent studies have shown that FF contains extracellular vesicles (EVs) [22,23]. EVs are membrane-bound biological nanoparticles released by almost every cell type [24]. There is a growing body of literature that describes EVs as mediators of intercellular communication [25,26]. Recent studies have shown that FF EVs play a vital and supportive role in various reproductive processes such as cumulus expansion [27] and meiotic resumption of oocytes [28], ovarian physiology [23], modulation of the oviduct in preparation for fertilization and embryo development [22]. 

EVs are known to affect spermatozoa’s functions; for example, prostasomes are a type of extracellular vesicles secreted by epithelial cells of the prostate that bind to the head of spermatozoa and help with their survival in the female reproductive tract. Prostasomes evoke motility of spermatozoa and help them to attain functional maturation, i.e., capacitation, which is eventually followed by the acrosome reaction [29,30]. Different investigation reports have shown that oviductal extracellular vesicles (oEVs) improve sperm motility and can keep the sperm acrosome intact [31,32]. 

In the current investigation, we hypothesized that EVs derived from bovine FF are capable of regulating key functional properties of spermatozoa, i.e., maintaining sperm viability and inducing capacitation and acrosome reaction. Our results support this hypothesis and show that FF EVs act as regulators of several functional properties of spermatozoa. This is an important observation, shedding light on a novel mechanism of spermatozoa function modulation, previously completely neglected and unknown. 

## 2. Materials and Methods

### 2.1. Collection of Bovine FF and Isolation of EVs

Collection of the FF and isolation of EVs were carried out following the protocol described earlier by our group with slight modifications [22,33]. In brief, ovaries were obtained from the slaughterhouse and washed three times with physiological saline. FF was aspirated from small (2–6 mm in diameter), medium (6–9 mm in diameter) and large follicles (>9 mm in diameter) using a vacuum pump (Minitüb GmbH, Tiefenbach, Germany) and pooled according to the follicle size category.

Isolation of EVs from the FF was carried out using benchtop size exclusion chromatography (SEC) columns. A total of 20 fractions was collected (each fraction 500 µL), of which fractions 1–4 (void volume) were discarded and fractions 5–7 were collected and pooled together as EV fractions. In addition, fractions 10–13 and 14–16 were collected and pooled separately as non-EV fraction-1 (Non-EV1) and non-EV fraction-2 (Non-EV2), respectively. All pooled fractions (EV and non-EV) were concentrated using Amicon^®^ Ultra-15 centrifugal filter devices (10 kDa cut-off, Merck Millipore Ltd., Tullagreen, Carrigtwohill, Co Cork Ireland) by centrifuging at 3000× *g* for 1 h at 4 °C. Collection and isolation of porcine FF EVs (pFF EVs) were carried out following the same protocol. 

### 2.2. Human Choriocarcinoma Cell Line (JAr) Cell Culture and Purification of JAr EVs

The human choriocarcinoma cell line (JAr) was acquired from ATCC^®^ (HTB-144 ™, Teddington, UK). JAr cells were cultured in RPMI 1640 media (Gibco, Paisley, Scotland) supplemented with 10% FBS, 1% L-glutamine and 1% Penicillin/Streptomycin at 5% CO_2_ and 37 °C. The culture media were changed every other day until cells reached 80% confluency. At the required confluency, the media were replaced by RPMI 1640 media (Gibco, Scotland) supplemented with 10% EV-depleted FBS, 1% L-glutamine and 1% Penicillin/Streptomycin and the JAr cells were further cultured for 24 h. Then, the conditioned media was collected for further processing. The isolation of EVs from JAr-conditioned media was performed according to the protocol published by Es-haghi et al., 2019 [34]. Briefly, the collected conditioned media was centrifuged at 400× *g* for 10 min to remove the cells. The supernatant was centrifuged again at 4000× *g* for 10 min and thereafter at 20,000× *g* for 15 min to remove cell debris and apoptotic bodies. To isolate EVs, conditioned media was concentrated to 500 µL with Amicon^®^ Ultra-15 centrifugal filter devices (10 kDa cut-off, Merck Millipore Ltd., Dublin, Ireland). EVs were isolated using the same SEC column as described before. EV-containing fractions 7–10 (fraction size 0.5 mL) were collected, pooled and concentrated using Amicon^®^ Ultra-15 centrifugal filter devices (10 kDa cut-off, Merck Millipore Ltd., Dublin, Ireland).

### 2.3. Nanoparticle Tracking Analysis (NTA)

The concentration and size profile of EVs were determined using a ZetaView PMX 100 NTA instrument (Particle Metrix GmbH, Ammersee, Bavaria, Germany) according to the standard manufacturer’s protocol with slight modification. In brief, using 100 nm particle size standards (Applied Microspheres BV, Leusden, The Netherlands. Catalogue no. 10100), the Zetaview^®^ was auto-aligned. The measurements of FF EVs were obtained in scatter mode under the following settings for every sample: sensitivity, -— 85; shutter speed, -— 70; frame rate, -— 30 fps; and number of cycles, -— 3. Each sample was measured in triplicate. To minimize inter-sample contamination the cell chamber of the instrument was washed between samples with Milli-Q^®^ followed by DPBS. 

### 2.4. Western Blot

Purified FF EVs and JAr EVs were concentrated to 300 μL using Amicon Ultra-15 centrifugal filter units (10 kDa cut-off, Merck Millipore Ltd., Dublin, Ireland). Subsequently, 100 μL of water, 100 μL of chloroform and 400 μL of methanol were added to each concentrated sample. The solutions were mixed by brief vortexing and centrifuged for 5 min at 14,000× *g* at RT. Three layers were formed, where a white precipitate represented the proteins. The top layer was carefully discarded and 400 μL of methanol was added to the precipitate and centrifuged at 14,000× *g* for 5 min. The pellets were air-dried and resuspended in 0.25% sodium dodecyl sulfate (SDS). FF and JAr EV samples were prepared in parallel. The JAr cell lysate was prepared by adding lysis buffer (150 mM NaCl, 1× protease inhibitors, 50 mM Tris-HCl, pH 7.5 and 1% Triton X-100) to JAr cells and incubating on ice for one hour. Samples were centrifuged for 5 min at 14,000× *g* at 4 °C and the protein-containing supernatant was collected. Protein concentrations were determined by the Quick Start™ Bradford Protein Assay (Bio-Rad, Berkeley, CA, USA). Either non-reducing or reducing Laemmli buffer was added to the protein samples and heated for 5 min at 95 °C. Samples were prepared in reducing Laemmli buffer for apoA-I detection and in non-reducing Laemmli buffer for CD63, CD9 and CD81 detection. Proteins were separated in twelve percent SDS-PAGE by following the standard protocol and transferred onto polyvinylidene difluoride membrane (Thermo Scientific, Rockford, IL, USA). For apoA-I detection, membranes were blocked in 5% nonfat dry milk in PBS-T (PBS + 0.05% Tween−20) and 5% BSA (Pan Biotech GmbH, Aidenbach, Germany) in PBS-T for CD63, CD9 and CD81 detection. Membranes were incubated with anti-apoA-I antibody (sc-376818, 1:1000, Santa Cruz Biotechnology Inc., Dallas, TX, USA), anti-CD63 antibody (ab68418, 1:500, Abcam, Cambridge, UK, or 556019, 1:1000, BD Biosciences, New Jersey, USA), anti-CD9 (sc-59140, 1:250, Santa Cruz Biotechnology Inc., Dallas, TX, USA) and anti-CD81 antibodies (555675, 1:500, BD Biosciences, NJ, USA) overnight at 4 °C, followed by incubation with goat anti-mouse secondary antibody (G21040, 1:20,000, Invitrogen, Thermo Fisher Scientific, Eugene, OR, USA) and horseradish peroxidase-conjugated goat anti-rabbit secondary antibody (G21234, 1:20,000, Invitrogen, Thermo Fisher Scientific, Eugene, OR, USA) for 1 h at RT. The membrane was washed three times with PBS-T after each incubation. Protein bands were detected using ECL SelectTM Western blotting detection reagent (GE Healthcare, Buckinghamshire, UK) with an ImageQuantTM RT ECL imager.

### 2.5. Transmission Electron Microscopy

EVs isolated using SEC (FF EVs and JAr EVs) were concentrated and deposited on Formvar-carbon-coated 200 mesh copper grids (Agar Scientific, Stansted, UK). The method described by Thery et al., 2018 [35], was used for transmission electron microscopy (TEM) analysis. In brief, EVs were fixed on grids in 2% paraformaldehyde (Sigma-Aldrich, Schnelldorf, Germany) and 1% glutaraldehyde (Polysciences, Warrington, PA, USA) before being contrasted in uranyl oxalate (a mixture of 4% uranyl acetate (Polysciences, Warrington, PA, USA) and 0.15 M oxalic acid (Sigma-Aldrich, Schnelldorf, Germany) and embedded in a mixture of methylcellulose (Sigma-Aldrich, Schnelldorf, Germany) and uranyl acetate (Polysciences, Warrington, PA, USA). Samples were observed with a JEM 1400 transmission electron microscope (JEOL Ltd. Tokyo, Japan) at 80 kV and digital images were acquired with a numeric camera (Morada TEM CCD camera, Olympus, Germany).

### 2.6. Washing of Spermatozoa

Frozen semen straws from three different bulls were processed separately following the methods described by our group previously [36]. In brief, frozen semen straws (4 straws from each bull) were thawed at 37 °C and the contents of the straws were layered over 4 mL of 60% isoosmotic Percoll^®^ solution (GE Healthcare, 17-0891-02, Vendevägen, Sweden) Subsequently, the overlayered semen samples were centrifuged at 300× *g* for 20 min at RT. This step was followed by washing the pellet with prewarmed EV-depleted sperm-TALP media and the samples were again centrifuged for another 5 min at 400× *g* at RT. The final pellets were then resuspended in pre-warmed sperm-TALP media and the concentrations of spermatozoa in samples were determined. 

### 2.7. Assessment of the Viability of Spermatozoa 

The viability of spermatozoa was assessed using the LIVE/DEAD^®^ Viability/Cytotoxicity Kit (MP 03224, ThermoFisher Scientific Inc., Santa Clara, CA, USA) by following the manufacturer’s protocol. In brief, 100 µL of freshly prepared working solution of EthD-1(4 µM) and calcein (2 µM) was added to 25 µL of sperm solution and mixed properly. The solution was incubated for 30 min at RT. After incubation, a smear of the sample was prepared on a microscope glass slide, covered with a coverslip and sealed. The viability of spermatozoa was examined under a fluorescent microscope. EthD-1- and calcein-labelled spermatozoa were classified into two categories as live spermatozoa and dead spermatozoa. Live spermatozoa displayed a green, fluorescent signal, whereas dead spermatozoa exhibited a red signal (Supplementary Figure S1A).

### 2.8. Assessment of Sperm Capacitation

The sperm capacitation process was evaluated using the method described by Fraser and McDermott, with slight modifications [37]. A chlortetracycline (CTC) working solution was prepared by dissolving CTC–HCl (Sigma-Aldrich, Saint Louis, MO, USA) with a final concentration of 750 µM in a buffer containing 20 mM Tris-HCl, 130 mM NaCl and 5 mM cysteine-HCl and the pH was adjusted to 7.0. The working solution was prepared freshly and wrapped in foil to prevent exposure to light and stored at 4 °C until further use. A spermatozoa–CTC solution was prepared by mixing 100 µL of sperm suspension and 100 µL of CTC working solution in a 1.5 mL wrapped tube. The microscope slides were prepared by adding 10 µL of the spermatozoa–CTC solution with a drop of 12.5% paraformaldehyde (pH 7.5) on a glass slide. To retard the fading of fluorescence, one drop of 0.22 M glycerol was added. The slides were covered with coverslips and examined for capacitation at 400× magnification using a fluorescent microscope. On average, 250–300 spermatozoa were analyzed per slide. CTC–HCl stained spermatozoa displayed three major fluorescence patterns: (I) Non-capacitated spermatozoa displayed a bright fluorescence in the entire sperm head, with or without a brighter equatorial band. (II) Capacitated spermatozoa showed a bright fluorescence in the acrosomal region of the sperm head, whereas the post acrosomal segment was non-fluorescent. (III) Acrosome-reacted spermatozoa had either a fluorescent or non-fluorescent post-acrosomal segment (Supplementary Figure S1B).

### 2.9. Assessment of Acrosomal Reaction

The acrosomal integrity of spermatozoa was assessed using the protocol described by Kitiyanant et al. with slight modifications [38]. Fluorescein isothiocyanate-Peanut agglutinin (FITC-PNA, Sigma-Aldrich, Saint Louis, MO, USA) and ethidium homodimer (EthD-1, Sigma-Aldrich, Saint Louis, MO, USA) were used to assess the acrosomal reaction. A total of 100 µL of spermatozoa and 100 µL of EthD-1 (4µm) were mixed properly in a 1.5 mL tube followed by incubation for 5 min at 38 °C. After 5 min of incubation, the excess EthD-1 was removed by adding 1 mL of DPBS and centrifuging the sample at 300× *g* for 5 min at 37 °C. The supernatant was discarded. A smear of the sample was prepared and air-dried. Then, the slide was fixed and permeabilized using 95% ethanol and 0.5% triton X-100, respectively, in the dark for 5 min. The slides were rinsed with DPBS and, subsequently, FITC-PNA (working solution 100 µg/mL) was added over the smear and incubated for 30 min at 38 °C in a moist and dark chamber. The slides were subsequently rinsed with DPBS, mounted with mounting media, covered with a coverslip and sealed. The morphology of spermatozoa for the acrosomal reaction was observed using a fluorescence microscope at 400× magnification and, on average, 250–300 spermatozoa were examined per slide. Bull spermatozoa were categorized into four categories based on the status of the observed acrosomal reaction: (I) Acrosome-intact spermatozoa showed bright fluorescence of acrosomal caps with a colorless post-acrosomal region. (II) Acrosome-reacting spermatozoa had bright fluorescence of acrosomal caps with patch-like fluorescence-stained equatorial segment. (III) Acrosome-reacted live spermatozoa displayed a patch-like fluorescence-stained equatorial segment and non-fluorescent acrosomal caps with clear post-acrosomal regions. (IV) Acrosome-reacted dead spermatozoa displayed fluorescence staining in the equatorial segment with non-fluorescence acrosomal caps and red post-acrosomal regions (Supplementary Figure S1C).

### 2.10. Surface Modification of EVs

EV surface proteins were cleaved using the protocol described by Skliar et al., 2017 [39]. In brief, 5 µL of 0.25% trypsin-EDTA was added to 30 µL of EV suspension and incubated for 20 min at 37 °C. To inactivate the trypsin, 5 µL of growth medium was added. Size profile, concentration and protein concentration of EVs were measured before and after the trypsin treatment.

### 2.11. Determination of Progesterone Concentration of FF and EVs

The chemiluminescence immunoassay-based measurement of the concentration of Progesterone in the FF and FF EV samples was conducted at the SYNLAB Eesti OÜ, Tartu, Estonia, using the ADVIA Centaur XP immunoassay system (Siemens Healthineers, Erlangen, Germany). The measuring range of the progesterone assay was 0.21–60 ng/mL (0.67–190.8 nmol/L).

### 2.12. Statistical Analysis

The data obtained from the assessments of viability, capacitation and acrosome reaction were analyzed using linear mixed models to ascertain the statistical significance of the observed differences between the supplementation groups. The use of linear mixed models was necessitated by the hierarchical structure of the data and, in the case of concentration gradient experiments, repeated measurements of the same samples. Linear mixed models (LMM) were fitted via the residual maximum likelihood (REML) approach. Unadjusted *p*-values were obtained from *t*-tests on the resulting estimated marginal means (EMMs) and were adjusted for multiple testing using the Bonferroni method. The paired Student’s *t*-test was used as the statistical test in the case of nanoparticle concentration data. The statistical analyses were conducted in R using packages lme4 [40] and emmeans [41] for linear mixed models. Graphs were produced with the ggplot2 [42] package in R and GraphPad prism 8.4.2.

### 2.13. Experimental Design

#### 2.13.1. Determining the Effects of FF EVs and Non-EV Fractions on the Viability, Capacitation and Acrosomal Reaction of Spermatozoa

In total, 5 million spermatozoa were incubated in 500 µL sperm-TALP media in 24-well culture plates with either EVs (5 × 10^8^ particles/mL) or 50 µL of concentrated non-EV fractions for 4 h. Control group spermatozoa were incubated for 4 h without supplementation. The effects of supplementing EV and non-EV fractions on spermatozoa were analyzed based on 3 outcome measures, sperm viability, capacitation and acrosomal reaction, at 0 and 4 h. The experiment was carried out on three different days with semen from three different bulls.

#### 2.13.2. Determining the Minimum Concentration of FF EVs Required for Modifying the Viability, Capacitation and Acrosomal Reaction of Spermatozoa

In total, 5 million spermatozoa were incubated in 500 µL sperm-TALP media in 24-well culture plates with different concentrations of large follicles FF EVs (in a range of 10 × 10^1^–10 × 10^8^ particles/mL) for 4 h. The outcome measures were analyzed at 0 and 4 h. The experiment was carried out on three different days with semen from three different bulls.

#### 2.13.3. Studying the Specificity of the Effects of FF EVs on the Maintenance of Sperm Viability, Induction of Capacitation and the Acrosomal Reaction of Spermatozoa

EVs from a different cellular source (a human choriocarcinoma cell line JAr and porcine follicular fluid-derived EVs (pFF EVs)) and differently sized bovine follicles (large, medium and small) were incubated with bovine spermatozoa. In total, 5 million spermatozoa were incubated in 500 µL sperm-TALP media (10 million/mL) in 24-well culture plates with EVs (5 × 10^6^ EVs in 500 µL TALP media) for 4 h. The effects of EV supplementation on the maintenance of sperm viability, induction of capacitation and acrosomal reaction were analyzed at 0 and 4 h. The experiment was carried out on three different days with semen from three different bulls.

#### 2.13.4. Studying the Effect of FF EV Surface-Modification on the Viability, Capacitation and Acrosomal Reaction of Spermatozoa

The surface of the FF EVs was modified by cleaving the surface proteins of EVs with trypsin as described earlier. In total, 5 million spermatozoa were incubated in 500 µL sperm-TALP media in 24-well culture plates with surface-modified EVs (5 × 10^6^ EVs in 500 µL TALP media) for 4 h. The concentration of EVs was adjusted for the supplementation as the concentration of EVs decreased after the trypsin treatment. The effects of the surface-modified EVs on sperm viability, capacitation and acrosomal reaction were analyzed at 0 and 4 h. The experiment was carried out on three different days with semen from three different bulls.

#### 2.13.5. Studying the Efficiency and Synergistic Effects of Progesterone and FF EVs on Sperm Viability, Capacitation and Acrosome Reaction

Based on our previous experiments, we concluded that FF EVs enhance the critical functions of spermatozoa. While the capacity of progesterone influencing the various functions of spermatozoa is already known, we aimed to study the cumulative effects of two active ingredients (progesterone and FF EVs) on spermatozoa. In order to study these effects, we designed an experiment with three different groups: spermatozoa incubated with FF EVs (5 × 10^6^ EVs in 500 µL TALP media); spermatozoa incubated with two different concentrations of progesterone (0.5 µg/µL and 1 µg/µL in 500 µL TALP media); spermatozoa incubated with EVs+progesterone and their respective controls for 4 h. The effects of FF EVs, progesterone and the combination of FF EVs + progesterone on sperm viability, capacitation and acrosome reactions were analyzed.

## 3. Results

### 3.1. Characterization of EVs

**Figure 1:**
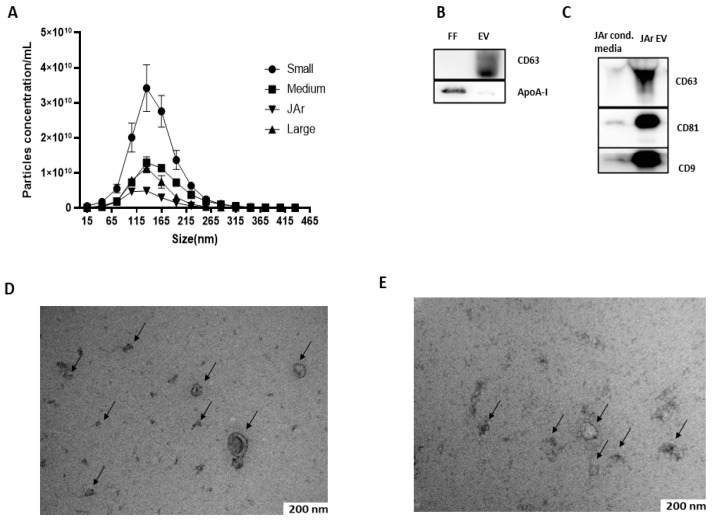
Characterization of FF EVs and JAr EVs. (**A**) Size profile and concentration of EVs measured by NTA. N = 3 of each of the sized follicles. Error bars display mean ± standard deviation (SD). (**B**) Western blot analysis of FF EVs for EV-specific marker. The presence of specific EV marker CD63 in EVs samples confirmed the successful isolation of EVs from FF. The apoA-I marker was used as a purity control for EVs and a strong signal of apoA-I was observed in unpurified FF samples compared to EVs, which indicates that EVs purified from FF by SEC had little or no contamination where ApoA-I indicates the purity of EVs. (**C**) EVs purified from JAr-conditioned medium showed a strong positive signal for EV-specific markers CD63, CD81 and CD9 compared to unpurified JAr-conditioned medium, which showed the enrichment of EVs compared to unpurified samples. (**D**) EVs purified from bovine FF were analyzed by TEM, where the black arrow indicates the typical cup-shaped of EVs. (**E**) EVs purified from JAr-conditioned medium were confirm and characterized by TEM, where the black arrow indicates the typical cup-shaped of EVs.

FF EVs and JAr EVs were characterized by nanoparticle tracking analysis (NTA), Western blot (WB) and transmission electron microscopy (TEM). Mostly, the nanoparticles as analyzed by NTA were less than 350 nm in size, with a large population in the 50–250 nm size range, which is the typical size range of EVs [43] NTA results suggest that the FF of small-sized follicles had a higher concentration of EVs than medium- and large-sized follicles (Figure 1A). Purified FF EVs and JAr EVs were analyzed by Western blot for specific EVs markers. Specific EVs markers were present in both FF EVs (CD63; Figure 1B) and JAr EVs (CD63, CD81, CD9; Figure 1C). Apolipoprotein A-I (apoA-I) was used as a marker for EV purity (Figure 1B). The TEM analysis visualized the presence of EVs with a typical cup-shaped morphological characteristic of EVs. Black arrows indicate the EVs (Figure 1D,E).

### 3.2. Effects of FF EV and Non-EV Fractions of SEC on the Viability of Spermatozoa

**Figure 2:**
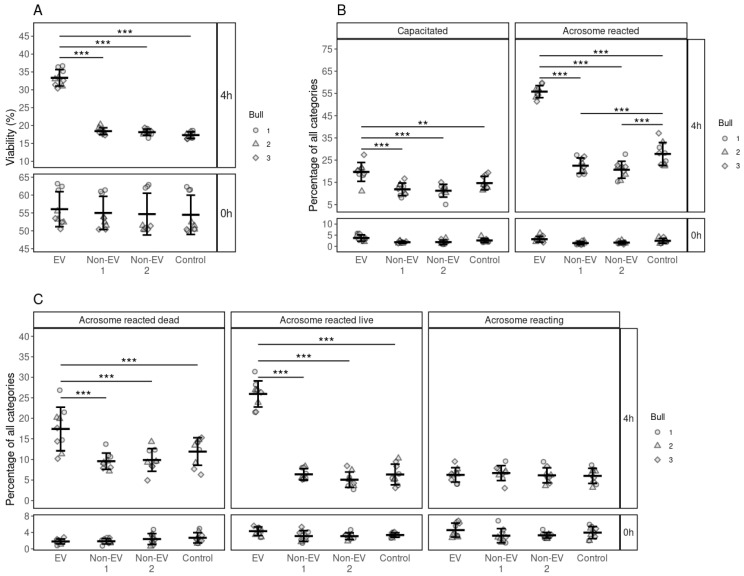
The effects of supplementing FF EV and non-EV fractions on the viability, capacitation and acrosome reaction of spermatozoa. (**A**) The viability of spermatozoa at 0 and 4 h in response to different supplementations. (**B**) The percentage of spermatozoa in the different stages of the capacitation process at 0 and 4 h after supplementation. (**C**) The percentage of spermatozoa in the different stages of acrosome reaction at 0 and 4 h after supplementation. Error bars display the mean ± standard deviation (SD). Different symbols represent measurements of samples from different bulls. Asterisks mark statistically significant differences among the supplementation groups and relate to *p*-values as follows: ** *p* ≤ 0.01; *** *p* ≤ 0.001.

Sperm viability was analyzed at 0 and 4 h after the EV supplementation (Figure 2). The overall percentage of live spermatozoa had declined across all groups from 55.05 ± 4.91%, mean ± SD, at 0 h to about 17.98 ± 0.86% after 4 h. However, average sperm viability was significantly higher in the EV-supplemented groups (33.35 ± 2.17, *p* ≤ 0.001) than Non-EV1, Non-EV2 and control groups (Figure 2A). Our results suggest that FF EVs support the viability of spermatozoa.

### 3.3. Effects of FF EV and Non-EV Fractions of SEC on the Capacitation and the Acrosome Reaction

Different stages of the capacitation processes of spermatozoa were analyzed at 0 and 4 h. At 0 h, most of the spermatozoa were non-capacitated (95.38 ± 1.55%) in all groups. After 4 h of incubation, most of the spermatozoa were non-viable and remained incapacitated in all the supplementation groups except the EV supplementation groups. The percentage of capacitated spermatozoa (19.68 ± 4.00%) with EV supplementation was significantly higher (*p* ≤ 0.001) than those in the non-EV1, non-EV2 and control groups (Figure 2B). We observed the same trend in the acrosome-reacted spermatozoa where EV supplementation significantly increased the incidence of acrosome reactions (55.83 ± 2.56%, *p* ≤ 0.001) compared to non-EV1, non-EV2 and control (Figure 2B).

In the case of acrosome reaction, at the start of incubation, most spermatozoa in all groups were acrosome-intact (90.57 ± 2.59%). After 4 h, the percentage of different stages of the acrosome reaction was similar across all groups, except that of the EV-supplemented group. Most of the spermatozoa incubated without EV supplementation remained acrosome-intact (77.04 ± 3.72%) after incubation for 4h. However, with EV supplementation, the percentage of acrosome-reacted dead spermatozoa (17.40 ± 4.99%, *p* ≤ 0.001) was significantly higher compared to the control, non-EV1 and non-EV2 supplemented groups. We observed the same effect in the case of acrosome-reacted live spermatozoa, where the percentage of acrosome-reacted live spermatozoa in the EV supplemented group was significantly higher (25.93 ± 2.99%, *p* ≤ 0.001) than in the control, non-EV1 and non-EV2 (Figure 2C). Our results indicate that EVs from FF increased the percentage of acrosome-reacted spermatozoa while maintaining the viability of spermatozoa.

### 3.4. Determining the Minimum Concentration of FF EVs Required to Improve the Viability, Capacitation and Acrosomal Reaction of Spermatozoa

**Figure 3:**
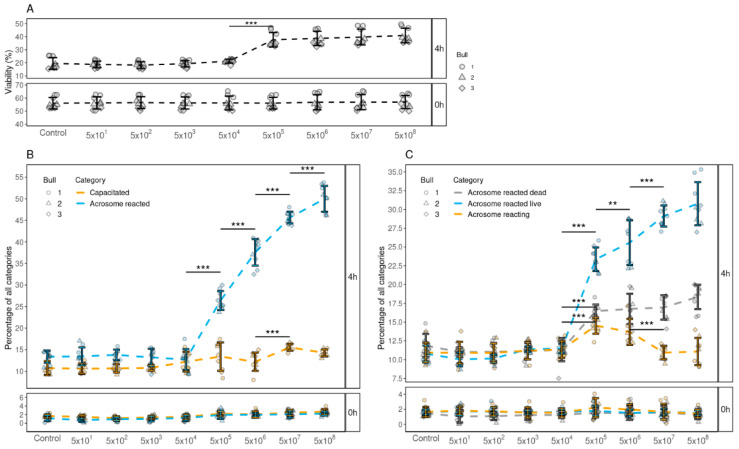
The effects of supplementing different concentrations of FF EVs (per 500 µL) on the viability, capacitation and acrosome reaction of spermatozoa. (**A**) The percentage of live spermatozoa supplemented with different EV concentrations at 0 and 4 h. (**B**) The percentages of spermatozoa at different stages of capacitation reaction 0 and 4 h after supplementation with different EV concentrations. (**C**) The percentages of spermatozoa in different stages of acrosomal reaction 0 and 4 h after supplementation. Error bars display the mean ± standard deviation (SD). In the background, different symbols represent measurements of samples from different bulls. Asterisks mark statistically significant differences between different concentrations and relate to p-values as follows: ** *p* ≤ 0.01; *** *p* ≤ 0.001. Whereas all pairwise comparisons were conducted, statistically significant differences are marked only for adjacent concentrations.

As the results from our previous experiments indicated that FF EVs support the viability, induction of capacitation and acrosome reaction of spermatozoa, our next objective was to determine the minimum number of EVs that are required to modify these various functional aspects. Our results indicated that a minimum of 1 × 10^6^ EVs were required to support sperm viability (Figure 3A) and enhance both capacitation (Figure 3B) and acrosomal reaction (Figure 3C) of spermatozoa. Moreover, our results also indicated that, with the increase in the number of EVs, the percentages of spermatozoa with enhanced viability, capacitation and acrosome reaction also increased. The group with the concentration of 1 × 10^9^ EVs displayed the highest percentages of viability, capacitation and acrosome-reacted spermatozoa.

### 3.5. The Specificity of the Effects of FF EVs on the Viability, Capacitation and Acrosomal Reaction of Spermatozoa

**Figure 4:**
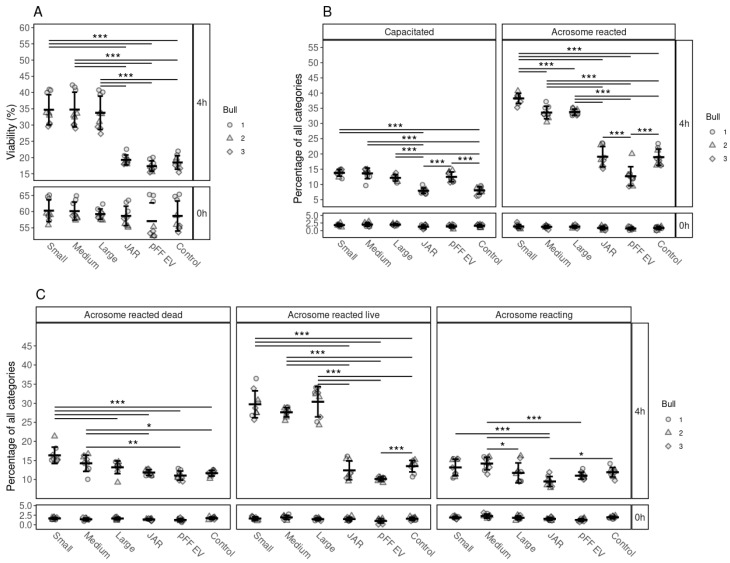
The effects of supplementation of EVs from different cellular sources on the viability, capacitation and acrosomal reaction of spermatozoa. (**A**) Percentages of live spermatozoa 0 and 4 h after supplementation with EVs from small, medium and large follicles, JAr EVs, pFF EVs and control. (**B**) Percentages of spermatozoa at different stages of capacitation reaction 0 and 4 h after supplementation with EVs from different sources. (**C**) Percentages of spermatozoa at different stages of the acrosomal reaction after supplementation with EV from different sources at 0 and 4 h. Error bars display the mean ± standard deviation (SD). Different symbols represent measurements of samples from different bulls. Asterisks mark statistically significant differences between the supplementation groups and relate to *p*-values as follows: * *p* ≤ 0.05; ** *p* ≤ 0.01; *** *p* ≤ 0.001. Double lines annotate *p*-values of the same category. In that case, asterisks above the top line also apply to the bottom line. The ends of the lines mark the groups that were contrasted.

Our results indicate that, regardless of the size of the follicle, all bovine FF EVs had a similar positive effect on sperm viability (Figure 4A). After 4 h of incubation, the percentages of live spermatozoa were significantly higher in FF EV-supplemented groups, than in those supplemented with non-FF EVs, JAr EVs and pFF EVs, which did not enhanced the viability of spermatozoa when compared to the control group.

We observed the same tendency for capacitation induction and acrosome reaction induction, where FF EVs had a significant positive effect compared to JAr EVs, pFF EVs and the control group (Figure 4B,C).

### 3.6. The Effect of Surface-Modified FF EVs on the Viability, Capacitation and Acrosomal Reaction of Spermatozoa

**Figure 5:**
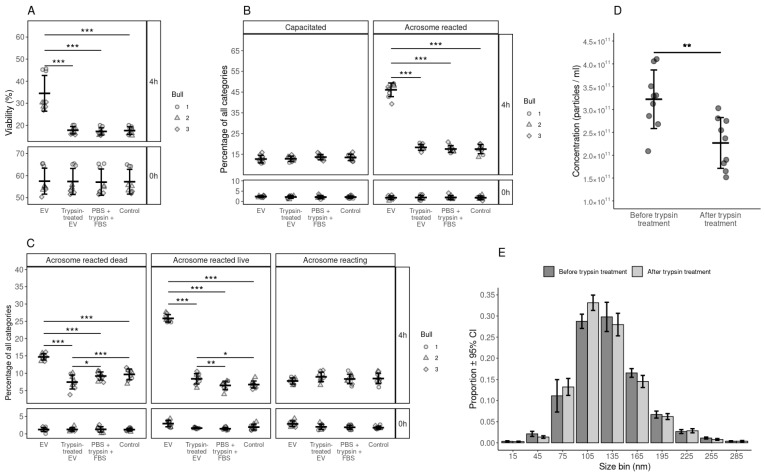
Analysis of functional aspects of bovine spermatozoa by incubating them with trypsin treated EVs, non treated EVs and PBS+trypsin+FBS and control. (**A**) Percentage of live spermatozoa at 0 and 4 hours after supplementation with treated and non treated EVs, FBS+trypsin+PBS and Control group. (**B**) Percentage spermatozoa in different stages of capacitation reaction at 0 and 4 hours after supplementation with treated and non treated EVs, FBS+trypsin+PBS and Control group. (**C**) The percentages of spermatozoa in different stages of the acrosomal reaction at 0 and 4 hours after supplementation with treated and non treated EVs, FBS+trypsin+PBS and Control group. (**D**) The concentration of nanoparticles in the EV samples before and after trypsin treatment. (**E**) Size profile of nanoparticles in the EV samples before and after trypsin treatment. Error bars represent 95% confidence intervals (CI). With the exception of subfigure E, error bars display the mean ± standard deviation (SD). Different symbols represent measurements of samples from different bulls, where applicable. Asterisks mark statistically significant differences and relate to p-values as follows: * *p* ≤ 0.05; ** *p* ≤ 0.01; *** *p* ≤ 0.001.

The objective of this experiment was to identify the effect of surface-modified EVs (trypsin-treated) on sperm viability, capacitation and acrosome reaction. The results showed that, after trypsin treatment, EVs no longer supported sperm viability (Figure 5A). We also observed that the EVs treated with trypsin lost their capacity to induce capacitation (Figure 5B) and the acrosome reaction (Figure 5C). Moreover, we observed a significant reduction in the total number of EVs after trypsin treatment (Figure 5D) and the number of large-sized EVs after trypsin treatment (Figure 5E). There was approximately an 82% reduction in the protein concentration after trypsin treatment (1.017 mg/mL before and 0.185 mg/mL after trypsin treatment).

### 3.7. The Efficiency and Synergistic Effects of Progesterone and FF EVs on Sperm Viability, Capacitation and Acrosome Reaction

**Figure 6:**
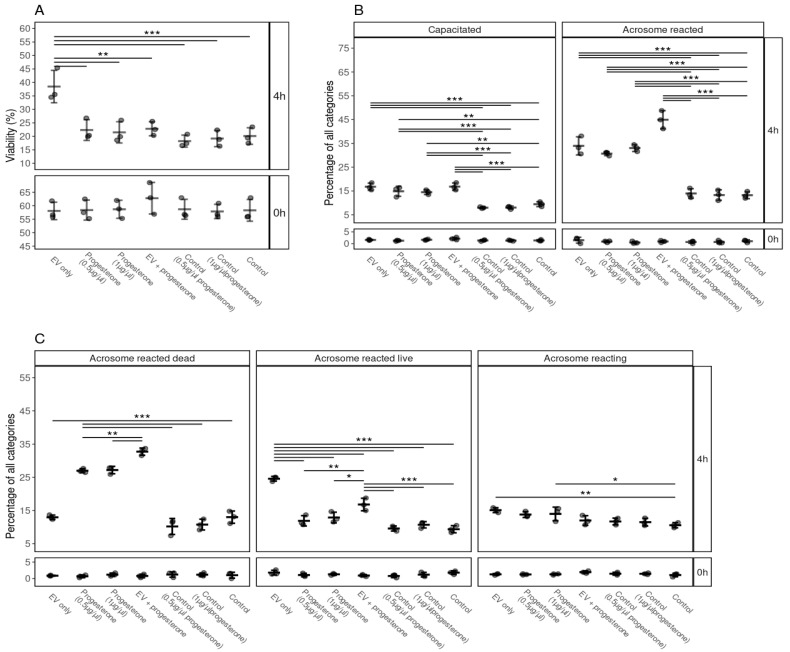
The effects of supplementation of EVs and different concentrations of progesterone and combination of FF EVs and progesterone on the viability, capacitation and acrosomal reaction of spermatozoa. (**A**) Percentages of live spermatozoa 0 and 4 h after supplementation with EVs, progesterone (0.5, 1 µg/µL), combination of FF EVs and progesterone, and controls. (**B**) Percentages of spermatozoa at different stages of capacitation reaction 0 and 4 h after with different supplementations. (**C**) Percentages of spermatozoa in different stages of the acrosomal reaction after supplementation with EV, different concentrations of progesterone and combinations of FF EVs and progesterone at 0 and 4 h. Error bars display the mean ± standard deviation (SD). Different symbols represent measurements of samples from different bulls. Asterisks mark statistically significant differences between the supplementation groups and relate to *p*-values as follows: * *p* ≤ 0.05; ** *p* ≤ 0.01; *** *p* ≤ 0.001. Double lines annotate *p*-values of the same category. In that case, asterisks above the top line also apply to the bottom line. The ends of the lines mark the groups that were contrasted.

Our results indicate that FF EVs and progesterone (0.5 and 1 µg/µL) induced capacitation and acrosome reactions (Figure 6B,C). Interestingly, the viability of spermatozoa was not supported by progesterone and it was significantly lower compared to EVs (Figure 6A). We also observed a significant difference in the case of acrosome-reacted live spermatozoa, where progesterone did not prolong the life span of spermatozoa after acrosome reaction (Figure 6C). A higher percentage of spermatozoa underwent capacitation and acrosomal reaction in the progesterone + FF EVs group (Figure 6B,C). Our results suggest that FF EVs and progesterone enhanced capacitation and the acrosomal reaction process in spermatozoa. However, FF EVs seemed to be more competent in supporting the viability of spermatozoa than progesterone and kept the spermatozoa alive after undergoing the process of acrosomal reaction.

### 3.8. The Concentration of Progesterone in FF and FF EVs

The concentration of progesterone in FF and FF EVs was determined using a chemiluminescence immunoassay system to identify if FF EVs had any progesterone. While progesterone was detected in FF, it was not detectable in FF EVs (Table 1).

The chemiluminescence immunoassay-based measurement of the concentrations of progesterone was performed using the ADVIA Centaur XP immunoassay system (Siemens Healthineers, Germany). The measuring range of the progesterone was 0.21–60 ng/mL (0.67–190.8 nmol/L).

## 4. Discussion

It has been demonstrated before that FF EVs mediate intercellular communication between female somatic cells and the oocyte. In this study, we show that EVs originating from the bovine FF are also able to mediate communication between the female somatic cells and male germ cells, i.e., spermatozoa. This is a novel dimension of communication between the females and the males that so far has been completely obscured. Although there are varying reports about the amount of FF released during ovulation and captured by the oviduct [15], nearly all studies are in agreement that, following ovulation, FF reaches the oviduct; therefore, it interacts with and affects sperm functions. The uncovering of the FF mode of communication through EVs widens our understanding of the collaboration between the mother and the progeny during the early stages of reproduction. This finding (involvement of FF EVs in the modulation of spermatozoa functions) is particularly important in unravelling the molecular mechanisms of events such as sperm competition and cryptic female choice [44].

In mammals, during mating, a large population of spermatozoa are deposited in the female reproductive tract. Spermatozoa need to travel to the upper parts of the female reproductive tract to the site of fertilization to meet and fertilize the egg. In order to fertilize the egg, spermatozoa need to undergo several physical and biomolecular changes, including hyperactivations, capacitation and acrosome reaction.

Only EVs obtained from bovine FF were able to maintain sperm viability, induce sperm capacitation and acrosome reaction. EVs obtained from other sources could not maintain sperm viability or induce capacitation and acrosome reaction in spermatozoa. This is an important aspect of the interaction of EVs with their responding cells. Some studies have reported that EVs produced by different cell types affect many different cells [45]. However, other studies showed that EVs produced by specific cells affect a particular responder cell or cell type and alter their functions, for example, cancerous cells affecting the function of the cells at the metastatic niche [25] or trophoblast EVs affecting the endometrial epithelial cells [34] during the embryo implantation process. Understanding the mechanisms that mediate the effects of FF EVs on spermatozoa may shed light on understanding general mechanisms that maintain such specificity of EVs function for specific responder cells. If the mechanisms mediating the specificity of EVs for specific responder cells is discovered, events beyond the prospects of reproductive physiology such as the role of EV in cancer metastasis may also be uncovered.

Our results show that EVs obtained from porcine FF had no effect on bull spermatozoa function, which could mean that FF EVs effects on spermatozoa are indeed species-specific. Understanding the mechanisms behind the maintenance of this species specificity will be important. So far, it is known that sperm binding to the zona pellucida, sperm penetration of the zona pellucida and sperm binding to oviductal cells [46] are species-specific events. The effect of FF EVs on the maintenance of sperm viability and induction of capacitation and acrosome reaction would be another layer of natural mechanisms that assures the species specificity at the time of fertilization and reproduction.

Different surface proteins or cargo carried by EVs could explain the mode of action and specificity of bovine FF EVs in transferring the required signals to bull spermatozoa [47,48]. We found that non-EV fractions of FF obtained from size exclusion chromatography did not positively affect the key functional properties of spermatozoa. The absence of EVs and other EV specific proteins or biomolecules in the non-EVs fractions might be the reason that these fractions had no effect on spermatozoa functional parameters. Moreover, progesterone concentration was measured in both unpurified FF and size exclusion chromatography-purified EV fractions. Progesterone was present in FF (collected from small, medium and large follicles) and was not present in the detectable range in the EV fractions purified from FF. Therefore, it seems that progesterone in FF EV preparations is either absent or in such negligible quantities that it cannot be the reason for the effect of EVs on spermatozoa observed in our experiments. Hence, the EVs and their cargo/surface proteins are probably responsible for the maintenance of the spermatozoa viability and induction of the capacitation and acrosomal reaction.

The results show that a minimum EV concentration of 5 × 10^5^ particles/500 µL was sufficient to induce significant changes in the aforementioned functional parameters of spermatozoa. We observed that EVs could maintain the total sperm viability about 15% higher than the control and other groups (after 4 h). It seems a single EV particle was sufficient to support the viability of a single spermatozoon. Nevertheless, we observed a concentration-dependent effect of EVs on sperm capacitation and acrosome reaction, which had a notable resemblance to enzyme–substrate reaction patterns. In the viability analysis, the curve reached a plateau and the effects did not increase any further after increasing the concentration of FF EVs beyond a certain EV concentration. A similar trend was discovered on the induction of sperm capacitation and acrosome reaction. A possible explanation for this EV concentration-dependent effect on spermatozoa might be that EVs work as enzymatic catalysts during capacitation and acrosome reaction processes. A series of biochemical changes occur in spermatozoa undergoing capacitation. This process helps spermatozoa acquire hypermotility and move towards the oocyte. The changes occurring during capacitation prime the spermatozoa for the acrosome reaction, which is an exocytotic event induced by a Ca^2+^ influx [49]. It plays an essential role during fertilization by making spermatozoa competent to penetrate the oocyte’s zona pellucida and fuse with the egg plasma membrane. All these processes involve substrate reactions and require additional energy [50]. Several reports have shown that EVs are involved in enzyme kinetics and are carriers of enzymes [51,52]. Hence, it could conceivably be hypothesized that FF EVs might be the carriers of some enzymes that contribute to the uplifting of spermatozoa viability, induction of capacitation and acrosomal reaction.

The percentage of acrosome-reacted live spermatozoa increased with the increase in the concentration of supplemented FF EVs. Previously, it was known that the binding of spermatozoa to the zona pellucida (ZP) of the oocyte triggers the acrosome reaction in spermatozoa. However, according to recent in vitro studies in mice, spermatozoa initiate their acrosome reaction before contact with the ZP [53]. In the female reproductive tract, after spermatozoa acquire functional maturation (capacitation), they undergo the acrosome reaction [54,55]. Usually, the life span of acrosome-reacted spermatozoa are short and they deteriorate fast. In our study, EVs might have triggered spermatozoa to undergo acrosome reaction faster while also prolonging the life span of the acrosome-reacted spermatozoa. Several studies have demonstrated that both acrosome-intact and acrosome-reacted spermatozoa are capable of binding to the ZP [56], penetrating it and fertilizing the egg(s) [57,58]. One may wonder whether spermatozoa that have undergone acrosome reaction as a result of being exposed to FF EVs are also capable of binding to the zona pellucida. Therefore, the intact acrosome spermatozoa might not necessarily be the initiators of oocyte interaction during fertilization.

The concentration and the molecular cargo of FF EVs may vary depending on the size of the ovarian follicle [59]. The size of the ovarian follicle is known to have a relationship with the competence of the oocyte [60] and future embryo development [61] during the assisted reproduction process. Nivet et al., 2016, when analyzing embryo development concerning the size of the follicle from which the individual oocyte was aspirated showed that oocytes from medium-sized follicles produced a higher number of transferable embryos. In another study, Wirleitner et al. [60] showed that the transfer of blastocysts derived from oocytes aspirated from small (<1 mm) follicles, in comparison to medium (1–6 mm) or large (>6 mm) follicles, tended to produce a higher live birth rate. While these differences could be attributed to the differences in FF EVs in differently sized follicles, a different degree of effects of EVs originating from differently size follicles on the key functions of spermatozoa was also expected. FF EVs collected from differently sized follicles were able to affect the sperm viability, capacitation and acrosomal reaction. However, higher percentages of spermatozoa underwent acrosome reactions when incubated with FF EVs collected from smaller-sized follicles. This result was obtained even though the supplemented quantities of FF-EVs in the three groups were similar. Thus, types of EVs present in small follicles may be more potent in supporting spermatozoa’s functional properties.

To understand the possible mechanism of EV-mediated effects on spermatozoa, we tried to modify the surface of FF EVs using trypsin to cause the breakdown of some proteins on the EV surface. Interestingly, trypsin-treated FF EVs lost their ability to trigger capacitation, acrosome reaction and induce viability in spermatozoa. There can be several explanations for this result; either the membrane proteins involved in EV uptake by spermatozoa were destroyed or trypsin-treated FF EVs lost the capacity to fuse with the sperm plasma membrane. Therefore, further investigations are required to understand the mechanism of action of FF EVs on spermatozoa.

It is well established that FF enhances the ability of spermatozoa to undergo capacitation, acrosomal reaction [1,62,63] and supports sperm motility [64]. Furthermore, previous studies have demonstrated that progesterone is the main active component of FF affecting different functional properties of spermatozoa such as capacitation and acrosome reaction [65]. Our study’s current findings suggested that FF EVs are competent in enhancing spermatozoa viability, capacitation and acrosome reaction. Based on these findings, we also studied the effect of progesterone and synergistic effects of progesterone and FF EVs on various functions of spermatozoa. This study indicated that both FF EVs and progesterone enhanced sperm capacitation and acrosome reaction separately. However, the cumulative effect of progesterone and FF EVs induced capacitation and acrosome reaction in a higher percentage of spermatozoa. This finding is consistent with that of [66], who also found that progesterone and FF exert a synergistic effect on zona pellucida-induced acrosomal reaction. One unanticipated finding was that FF EVs were more capable of maintaining sperm viability and prolonging spermatozoa life span after acrosome reaction than progesterone alone. It can be speculated that a synergy existed in vivo where both progesterone and FF EVs contribute to maintaining the essential functions of spermatozoa.

Insemination of human spermatozoa with human FF increased the pregnancy rate in patients undergoing intrauterine insemination (IUI) [67] and in vitro fertilization (IVF) [68]. Our results showed that FF EVs enhance the viability, capacitation and acrosome reaction of frozen-thawed spermatozoa. More information regarding cargo/surface proteins of FF EVs would be of great importance to improve the current ART protocols that can eventually lead to successful fertilization. Further research is required to study the composition of FF EV cargo/surface responsible for causing these changes in the functional attributes of the spermatozoa. The results could be translated for therapeutic interventions when using assisted reproductive technologies.

## 5. Conclusions

Overall, the findings of our study demonstrated that FF EVs enhance the capacitation and acrosome reaction of spermatozoa along with maintaining their viability, all of which are of paramount importance for successful fertilization. These effects are specific to FF as the source of the EVs. We infer that the role of FF is not only to nourish the oocyte but that it also plays a key role in boosting the functional parameters of spermatozoa mediated via EVs. This can indeed be considered another important aspect of the interaction between different sexes. As FF EVs originate from female follicular somatic cells and oocytes, these EVs start their interaction with spermatozoa before the male gamete reaches the female gamete. Hence, they are probably also involved in modulating processes such as sperm competition and female cryptic choice. This will open another completely new era for understanding and explaining the molecular events taking place during the interactions between the sexes in the earliest stages of reproduction with significant consequences for our understanding of the physiology of reproduction and different aspects of evolutionary biology.

## Figures and Tables

**Table 1:** The concentration of progesterone in FF and FF-derived EV samples.

Sample Type	Concentration (nmol/L)
Follicular fluid (small)	9.63 nmol/L
Follicular fluid (medium)	6.64 nmol/L
Follicular fluid (large)	7.64 nmol/L
Follicular fluid EVs (small)	<0.67 nmol/L
Follicular fluid EVs (medium)	<0.67 nmol/L
Follicular fluid EVs (large)	<0.67 nmol/L

## Data Availability

Not applicable.

## Supplementary Materials

The following are available online at https://www.mdpi.com/article/10.3390/biology10111154/s1, Figure S1: Assessment methods of spermatozoa viability, capacitation and acrosome reaction (A) Viability assessment of bull spermatozoa where the green fluorescent-labelled spermatozoa were considered as live spermatozoa and the ones with a red fluorescent label represent dead spermatozoa; (B) different categories of capacitation status of bull spermatozoa stained with CTC-HCL: (I) non-capacitated spermatozoa, (II) capacitated spermatozoa and (III) acrosome reacted spermatozoa; (C) different categories of the acrosomal reaction status of bull spermatozoa stained with FITC-PNA and EthD-1: (I) acrosome-intact spermatozoa, (II) acrosome-reacting spermatozoa, (III) acrosome-reacted live spermatozoa and (IV) acrosome-reacted dead spermatozoa.

## Abbreviations

FF, follicular fluid; EVs, extracellular vesicles; pFF, porcine follicular fluid; NTA, nanoparticle tracking analysis; WB, Western blot; TEM, transmission electron microscopy; EthD-1, Ethidium homodimer; JAr, human choriocarcinoma cell line; CTC, Chlortetracycline; LMM, linear mixed models; IVF, in vitro fertilization; IUI, intrauterine insemination.
