# Supplementary material for: Bovine Follicular Fluid Derived Extracellular Vesicles Modulate the Viability, Capacitation and Acrosome Reaction of Bull Spermatozoa
Source: Biology (Basel). 2021 Nov 9;10(11):1154. doi: 10.3390/biology10111154 (PMC8614796; doi:10.3390/biology10111154)
Supplement: Supplementary file 1 [file biology-10-01154-s001.zip › biology-1413067-supplementary.pdf]

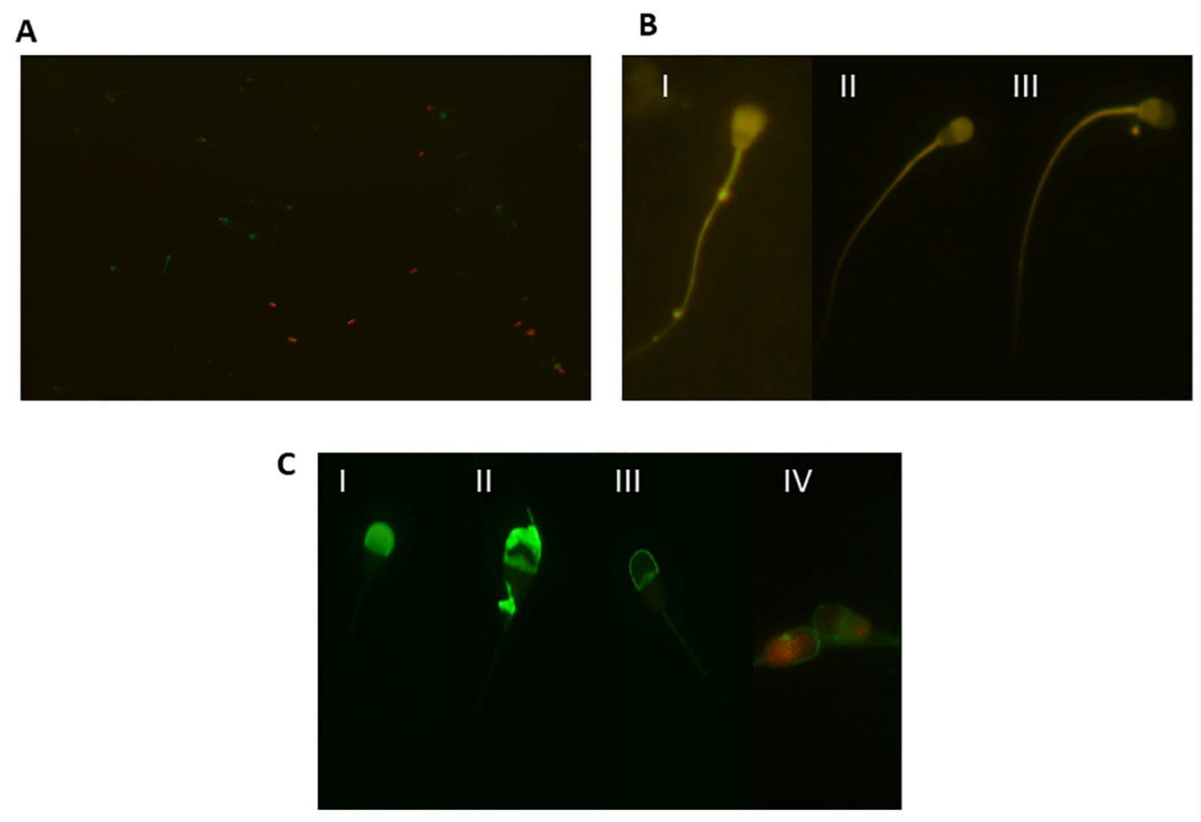

Supplementary figure S1. Assessment methods of spermtozoa viability,capacitation and acrosome reaction (A) Viability assessment of bull spermatozoa where the green fluorescent labelled spermatozoa were considered as live spermatozoa and the ones with red fluorescent label spermatozoa represent dead spermatozoa. (B) Different categories of capacitation status of bull spermatozoa stained with CTC-HCL. (I) Non-capacitated spermatozoa (II) capacitated spermatozoa and (III) acrosome reacted spermatozoa. (C) Different categories of the acrosomal reaction status of bull spermatozoa stained with FITC-PNA and EthD-1. (I) acrosome intact spermatozoa, (II) acrosome-reacting spermatozoa, (III) acrosome reacted live spermatozoa, and (IV) acrosome reacted dead spermatozoa.
